# Sparse Blind Spherical Deconvolution of diffusion weighted MRI

**DOI:** 10.3389/fnins.2024.1385975

**Published:** 2024-05-22

**Authors:** Clément Fuchs, Quentin Dessain, Nicolas Delinte, Manon Dausort, Benoît Macq

**Affiliations:** ^1^Institute of Information and Communication Technologies, Electronics and Applied Mathematics (ICTEAM), UCLouvain, Louvain-la-Neuve, Belgium; ^2^Institute of NeuroScience, UCLouvain, Brussels, Belgium

**Keywords:** diffusion MRI, spherical deconvolution, white matter, microstructure, multi-fascicle models

## Abstract

Diffusion-weighted magnetic resonance imaging provides invaluable insights into *in-vivo* neurological pathways. However, accurate and robust characterization of white matter fibers microstructure remains challenging. Widely used spherical deconvolution algorithms retrieve the fiber Orientation Distribution Function (ODF) by using an estimation of a response function, i.e., the signal arising from individual fascicles within a voxel. In this paper, an algorithm of blind spherical deconvolution is proposed, which only assumes the axial symmetry of the response function instead of its exact knowledge. This algorithm provides a method for estimating the peaks of the ODF in a voxel without any explicit response function, as well as a method for estimating signals associated with the peaks of the ODF, regardless of how those peaks were obtained. The two stages of the algorithm are tested on Monte Carlo simulations, as well as compared to state-of-the-art methods on real *in-vivo* data for the orientation retrieval task. Although the proposed algorithm was shown to attain lower angular errors than the state-of-the-art constrained spherical deconvolution algorithm on synthetic data, it was outperformed by state-of-the-art spherical deconvolution algorithms on *in-vivo* data. In conjunction with state-of-the art methods for axon bundles direction estimation, the proposed method showed its potential for the derivation of per-voxel per-direction metrics on synthetic as well as *in-vivo* data.

## 1 Introduction

Diffusion-weighted magnetic resonance imaging (DW-MRI) (Merboldt et al., 1985; Taylor and Bushell, 1985) is widely used for the study of tissue microstructure and fiber orientation. However, accurate *in-vivo* characterization of the complex and heterogeneous composition of nervous tissue remains challenging. Diffusion tensor imaging (DTI) (Basser et al., 1994) is one of the first and most commonly used diffusion model, mainly due to its ease of implementation, which only requires the acquisition of six DW-MRI images along with an additional non-weighted image. However, its underlying assumptions, particularly the presumption of Gaussian diffusion, restrict its capacity to characterize complex microstructures accurately. Following, more advanced models have been developed, trying to overcome some of the limitations of DTI, such as high angular resolution diffusion imaging (HARDI) together with Q-ball imaging (Tuch, 2004; Anderson, 2005). Q-ball imaging uses a reconstruction technique of the diffusion Orientation Distribution Function (dODF) based on the Funk-Radon Transform. As another possibility, Spherical Deconvolution (SD) (Healy et al., 1998; Tournier et al., 2004) uses a response kernel estimated from the DW-MRI data to perform the reconstruction of the ODF, later improved with a positivity constraint (Tournier et al., 2007) as well as a generalization to multiple shells and tissues (Jeurissen et al., 2014). A general formulation of the deconvolution problem was presented in Jian and Vemuri (2007) together with a comparison of various methods for solving it. Numerous additional methods were proposed and compared in Canales-Rodrguez et al. (2019). Especially, a sparse Bayesian learning framework, in which sparsity of the reconstructed ODF is promoted by assigning different variances to each entry of the function together with a zero mean Gaussian prior, has been proposed (Pisharady et al., 2018). It has the particularity of using a ball-and-stick based dictionary in order to retrieve diffusivity parameters in addition to orientations. More recently, ODF fingerprinting (Baete et al., 2019) as well as deep learning techniques based on rotationally equivariant layers were proposed (Elaldi et al., 2021, 2024) in order to improve the separability power of SD. Although Elaldi et al. (2021) highlights the formulation of the ODF reconstruction problem as *blind* deconvolution, a single response function is still used to train the deep neural networks in an unsupervised manner.

Consequently, SD algorithms have been somewhat limited in extracting additional signal features useful for estimating microstructural parameters such as diffusivity (in- or extra-axonal), although the ODF amplitude does contain information about parameters such as the fiber volume fraction (Raffelt et al., 2012). In order to recover per-voxel impulse responses as well as longitudinal and transverse diffusion coefficients, the Spherical Means Technique (SMT) was introduced in Kaden et al. (2016b) and generalized to multiple compartments in Kaden et al. (2016a). It uses an analytical model of the diffusion signal arising from an axon segment and leverages the invariance to the ODF of the mean signal of a voxel, provided that the response function is the same for all directions. Under similar assumptions, Anderson (2005) estimated per-voxel impulse responses in a constrained spherical deconvolution framework. It was later improved in Schultz and Groeschel (2013) with an ℓ_1/2_ regularization and better estimation of the mean diffusivity of the per-voxel impulse responses. Comparably, other methods, either based on Monte-Carlo simulations or on compartment models were proposed to compute microstructural metrics or diffusion properties. Notable contributions in this domain include works by Zhang et al. (2012); Panagiotaki et al. (2014); Scherrer et al. (2016); Nedjati-Gilani et al. (2017); Rensonnet et al. (2019); Palombo et al. (2020), and De Almeida Martins et al. (2021).

In this paper, an algorithm of *blind* spherical deconvolution is proposed, which does not need an explicit response function nor a generative model of the signal arising from the white matter, and retrieves information about *both* the orientations as well as the per-voxel per-direction impulse responses of the axon bundles, under the assumption that the observed DW-MRI signal is a sparse sum of axially symmetric signals each with its own orientation. This is performed by exploiting the relationships between Spherical Harmonics (SH) and D-Wigner functions and reformulating the deconvolution problem: the rotation of a signal axially symmetric around a basis axis can be expressed as a convolution with a *rotation filter* which is not dependent on the observed signal but only on mathematical properties of the SHs and D-Wigner functions. First, the theory underlying the proposed algorithm is explained. Then, two experiences, one on synthetic data obtained with Monte Carlo simulation, and the other on *in-vivo* data, are presented. Finally, the results and contribution of this work to existing spherical deconvolution algorithms are discussed.

## 2 Theory

For a complete definition of the notations and conventions used in this work, see Appendix A. In this section, we derive a reformulated deconvolution problem under a sparsity assumption using SHs and D-Wigner functions. For this, we study the relationship between a convolution operator on the sphere and rotations of signals axisymmetric around $\vec{u_{z}}$.

### 2.1 Convolution on the sphere

There is a conceptual difficulty in the definition of a convolution operator on the sphere. Indeed, on the 2D plane the value of the convolution at point **M** can be described as the correlation of the flipped convolution kernel with the underlying function values around **M**. In order to define a convolution on the sphere, the straightforward analogy is to consider rotations as a substitute for translations. Therefore, the result is defined over the set of rotations *SO*(3), elements of which can be represented by the three Euler angles, instead of the unit sphere *S*_2_. This conceptual difficulty led to numerous convolution operators on the sphere to be introduced in the literature, depending on the application (McEwen et al., 2007; Kennedy et al., 2011; Wei et al., 2011; Roddy and McEwen, 2021).

The convolution operator at the heart of the SD (Tournier et al., 2004), CSD (Tournier et al., 2007) and MSMT-CSD (Jeurissen et al., 2014) algorithms was introduced in Healy et al. (1998) and is defined as :


$$\begin{array}{l} \forall h\in L^{2}\left(SO\left(3\right)\right),\forall g\in L^{2}\left(S^{2}\right),\forall \omega \in S^{2},\\ \left(h*g\right)\left(\omega \right)=\int_{u\in SO_{3}}h\left(u\right)g\left(u^{-1}\omega \right)du. \end{array}$$

In the above equation, *SO*(3) is the set of three dimensional rotations and *S*^2^ the three dimensional 2-sphere $\{\omega \in {\mathbb{R}}^{3};||\omega ||{\;}_{2}=1$ }. *L*^2^(*S*^2^) and *L*^2^(*SO*(3)) are the set of square-integrable functions respectively defined over *S*^2^ and *SO*(3).

A theorem similar to the classical convolution theorem holds (Healy et al., 1998):


(1) $$\begin{array}{l} \forall h\in L^{2}\left(SO_{3}\right),\forall g\in L^{2}\left(S^{2}\right),{\left(h*g\right)}_{n}^{m}=\overset{n}{\underset{j=-n}{\sum }}h_{n}^{m,j}g_{n}^{j}, \end{array}$$

where $\forall n\in \mathbb{N},\forall m\in \left[\left|-n,n\right|\right],g_{n}^{m}$ are the coefficients of the expansion of *g* over the SHs $Y_{n}^{m}$ and $\forall n\in \mathbb{N},\forall m\in \left[\left|-n,n\right|\right],\forall j\in \left[\left|-n,n\right|\right],h_{n}^{m,j}$ are the coefficients of the expansion of *h* over the D-Wigner functions $D_{n}^{m,j}$.

### 2.2 Rotation of an axially symmetric signal

Let *C* a signal axisymmetric around $\vec{u_{z}}$ as well as antipodally symmetric. *C* can be interpreted as the response function of a parallel bundle of fibers, such as the response function of the CSD algorithm. This implies that $\forall m\neq 0,\forall n,n\text{odd},C_{n}^{m}=0$. Therefore, we have the SH expansion:


$$C\left(\omega \right)=\overset{\infty }{\underset{n=0}{\sum }}C_{n}^{0}Y_{n}^{0}\left(\omega \right).$$

A fundamental property of the SHs gives the expression of a rotated SH as a linear combination of the SHs of the same degree, where the coefficients of the combination can explicitly be derived with the D-Wigner functions $D_{n}^{m,j}$ (Healy et al., 1998):


$$\begin{array}{l} \forall n\in \mathbb{N},\forall j\in \left[\left|-n,n\right|\right],\forall u\in SO\left(3\right),\forall \omega \in S^{2},\\ \;Y_{n}^{j}\left(u^{-1}\omega \right)=\overset{n}{\underset{m=-n}{\sum }}D_{n}^{m,j}\left(u\right)Y_{n}^{m}\left(\omega \right). \end{array}$$

Therefore, the rotation of *C* by **u** ∈ *SO*(3), noted *C*_**u**_ can be computed as:


(2) $$\begin{array}{l} \;\forall u\in SO\left(3\right),\forall \omega \in S^{2},\\ C_{u}\left(\omega \right)=C\left(u^{-1}\omega \right)=\overset{\infty }{\underset{n=0}{\sum }}\overset{n}{\underset{m=-n}{\sum }}C_{n}^{0}D_{n}^{m,0}\left(u\right)Y_{n}^{m}\left(\omega \right). \end{array}$$

The SHs are related to the D-Wigner functions by Healy et al. (1998)


$$Y_{n}^{m}\left(\omega \right)=\sqrt{\frac{2n+1}{4\pi }}\bar{D_{n}^{m,0}}\left(\omega \right).$$

In the above equation, $\bar{D_{n}^{m,0}}\left(\omega \right)$ denotes the complex conjugate of the complex number $D_{n}^{m,0}\left(\omega \right)$. Although the $D_{n}^{m,0}$ are not defined over the unit sphere, consider that the $D_{n}^{m,0}$ is not dependent on the third Euler angle γ and that for a given **u** ∈ *SO*(3) represented by (*α*, *β*, 0), **u** can be seen as a point of *S*^2^ with coordinates *θ* = *β* and Φ = *α*.

Thus, Equation 2 becomes:


$$\forall u\in SO\left(3\right),\forall \omega \in S^{2},C\left(u^{-1}\omega \right)=\overset{\infty }{\underset{n=0}{\sum }}C_{n}^{0}\overset{n}{\underset{m=-n}{\sum }}\alpha_{n}\bar{Y_{n}^{m}}\left(u\right)Y_{n}^{m}\left(\omega \right),$$

where $\alpha_{n}=\sqrt{\frac{4\pi }{2n+1}}$.

Consequently, *C*_**u**_ can be rewritten with the convolution operator defined in Healy et al. (1998) (see Equation 1) as:


$$C_{u}=\left(U_{u}*C\right),$$

where *U*_**u**_ is defined by $\forall \omega \in S^{2},U_{\text{u}}\left(\omega \right)=\overset{\infty }{\underset{n=0}{\sum }}\overset{n}{\underset{m=-n}{\sum }}\alpha_{n}\bar{Y_{n}^{m}}\left(\text{u}\right)Y_{n}^{m}\left(\omega \right)$.

As in the foundational paper of Spherical Deconvolution (Tournier et al., 2004), this relationship is better visualized by regrouping coefficients of a same degree *n* in a vector:


(3) $$\begin{array}{l} C_{u}^{n}=C_{0}^{n}U_{u}^{n}, \end{array}$$

where both $C_{\text{u}}^{n}$ and $U_{\text{u}}^{n}$ are vectors of length 2*n*+1. It is to be noted that $U_{\text{u}}^{n}$ can be explicitly computed given the rotation **u**: this is a core idea that will allow us to perform *Blind* Spherical Deconvolution, i.e. perform the retrieval of *both* $U_{\text{u}}^{n}$ and $C_{n}^{0}$ at each voxel for each direction. This means that contrary to the CSD algorithm, in this work a different impulse response will be estimated for each axon bundle directions in every voxels. Other methods, such as the Spherical Means Technique (SMT) (Kaden et al., 2016b), have been proposed to estimate per-voxel impulse responses.

### 2.3 Linear superposition

The linear superposition of signals arising from intertwined axon bundles is assumed, as is common in DW-MRI (Tournier et al., 2013; Rensonnet, 2019).

Therefore, the DW-MRI signal *S* at a given voxel is considered to be a linear superposition of *K* signals *C*_*k*,_**u**__*k*__, each being obtained by rotation of a canonical signal *C*_*k*_ axially symmetric around $\vec{u_{z}}$, i.e.:


$$S\left(\omega \right)=\overset{K}{\underset{k=1}{\sum }}\nu_{k}C_{u_{k}}\left(\omega \right)=\overset{K}{\underset{k=1}{\sum }}\nu_{k}C_{k}\left(u_{k}^{-1}\omega \right),$$

where the ν_*k*_ are the mix coefficients and verify $\overset{K}{\underset{k=1}{\sum }}\nu_{k}=1$.

Equation 3 gives the SH expansion formulation:


(4) $$\begin{array}{l} S^{n}=\overset{K}{\underset{k=1}{\sum }}\nu_{k}C_{k,n}^{0}U_{u_{k}}^{n}. \end{array}$$

Equation 4 shows that at each degree *n*, the vector of coefficients of the SH expansion of the observed signal is a linear mix of *K* vectors $U_{{\text{u}}_{k}}^{n}$. The per-direction responses are given by the $\nu_{k}C_{k,n}^{0}$, while the sparse ODF, with exactly *K* directions for which it is non-zero, is given by the $U_{{\text{u}}_{k}}^{n}$.

### 2.4 Summary of the proposed algorithm

**Figure 1:**
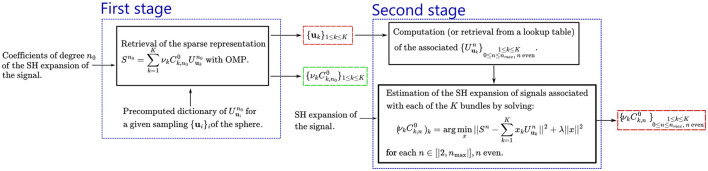
Graphical summary of the proposed algorithm, named Sparse Blind Spherical Deconvolution (SBSD). The first stage estimates the orientations of the fiber bundles, and the second stage the SH expansion of each bundle. Both stages are independent, and any orientation estimate can be used as an input of the second stage. The main outputs are highlighted in red and are the bundles orientations ({_**u**_*k*_}1 ≤ *k* ≤ *K*_) as well as their SHs expansion (${\left\{\nu_{k}C_{k,n}^{0}\right\}}_{\begin{array}{c} 1\leq k\leq K\\ 0\leq n\leq n_{max},\;n\text{even} \end{array}}$).

The algorithm presented in this paper, and summarized in Figure 1, is the retrieval of those vectors through a complex Orthogonal Matching Pursuit (OMP) algorithm (Tropp and Gilbert, 2007; Fan et al., 2012) for a chosen degree *n*_0_, and then the retrieval of the coefficients $C_{k,n}^{0}$ for all degrees *n* using the orientations found with the OMP. This approach will be refered to as **Sparse Blind Spherical Deconvolution (SBSD)** in the remainder of the work. The OMP retrieves a sparse representation of $S^{n_{0}}$ from prior knowledge of a precomputed dictionary


$$D^{n_{0}}=\left(U_{{\text{v}}_{1}}^{n_{0}}U_{{\text{v}}_{2}}^{n_{0}}...U_{{\text{v}}_{N_{R}}}^{n_{0}}\right)\in M_{2n_{0}+1,N_{R}}\left(\mathbb{C}\right),$$

of convolution filters associated with rotations **V** = {**v**_1_, **v**_2_, ..., **v**_*N*_*R*__} at degree *n*_0_. The OMP was chosen because the correlation between $U_{{\text{v}}_{1}}^{n_{0}}$ and $U_{{\text{v}}_{2}}^{n_{0}}$ quickly diminishes as the angular distance between **v**_1_ and **v**_2_ grows. The latter can be performed given any estimation {_**v**_*k*_}1 ≤ *k* ≤ *K*_ of the orientations, such as the peaks of the MSMT-CSD FOD, by solving


(5) $$\begin{array}{l} {\left(\nu_{k}C_{k,n}^{0}\right)}_{k}=\arg\underset{x}{\min}||S^{n}-\overset{K}{\underset{k=1}{\sum }}x_{k}U_{{\text{v}}_{k}}^{n}||{\;}^{2}+\lambda {\left(n\left(n+1\right)\right)}^{2}||x||{\;}^{2}. \end{array}$$

Equation 5 makes use of the regularization proposed in Descoteaux et al. (2007), with the *n*^th^ eigenvalue of the Laplace-Beltrami operator −*n*(*n*+1). The estimated coefficients are then used to reconstruct the per-voxel per-direction impulse responses $R_{k}\left(\omega \right)=\overset{N}{\underset{n=1}{\sum }}\nu_{k}C_{k,n}^{0}Y_{n}^{0}\left(\omega \right)$. Note that for *n* = 0, the matrix $\left(\begin{array}{c} U_{{\text{v}}_{0}}^{0}...U_{{\text{v}}_{K}}^{0} \end{array}\right)$ is singular, due to the infinite possible combinations for attributing mean values to the estimated impulses while exactly reconstructing the observed signal. Therefore, the mean of the diffusion signal is first subtracted from itself and this zero-mean signal fitted with coefficients of degree >0. Then, for each estimated impulse, the coefficient of degree 0 is given the value of the opposite of its minimum so that the response has positive values.

## 3 Methods

### 3.1 Experience A: validation on Monte Carlo simulations

A set *C* of 50 canonical signals was generated using Monte-Carlo simulations with the open-source MC-DC simulator (Rafael-Patino et al., 2020), using a substrate of cylinders oriented along $\vec{u_{z}}$ with gamma distributed radii, with all possible combinations of parameters values intra-axonal diffusivity $D_{in}\in \left\{1.5,2,2.25,2.5,3\right\}\times 10^{-9}{\text{m}}^{2}{\text{.s}}^{-1}$, extra-axonal diffusivity $D_{ex}\in \left\{1,1.5,2,2.5,3\right\}\times 10^{-9}{\text{m}}^{2}{\text{.s}}^{-1}$ and fiber volume fraction *fvf* ∈ {0.7, 0.8}. For all simulations, the gamma law was parametrized with α = 1.5 μm and β = 0.5. The simulated acquisition schemes have sampling of the sphere obtained with a Coulomb repulsion algorithm , with 100, 120, 130, 150or300 directions on the sphere for each b-value *B* ∈ {2000, 3000, 4000, 5000, 10000}s.mm^−2^.

**Figure 2:**
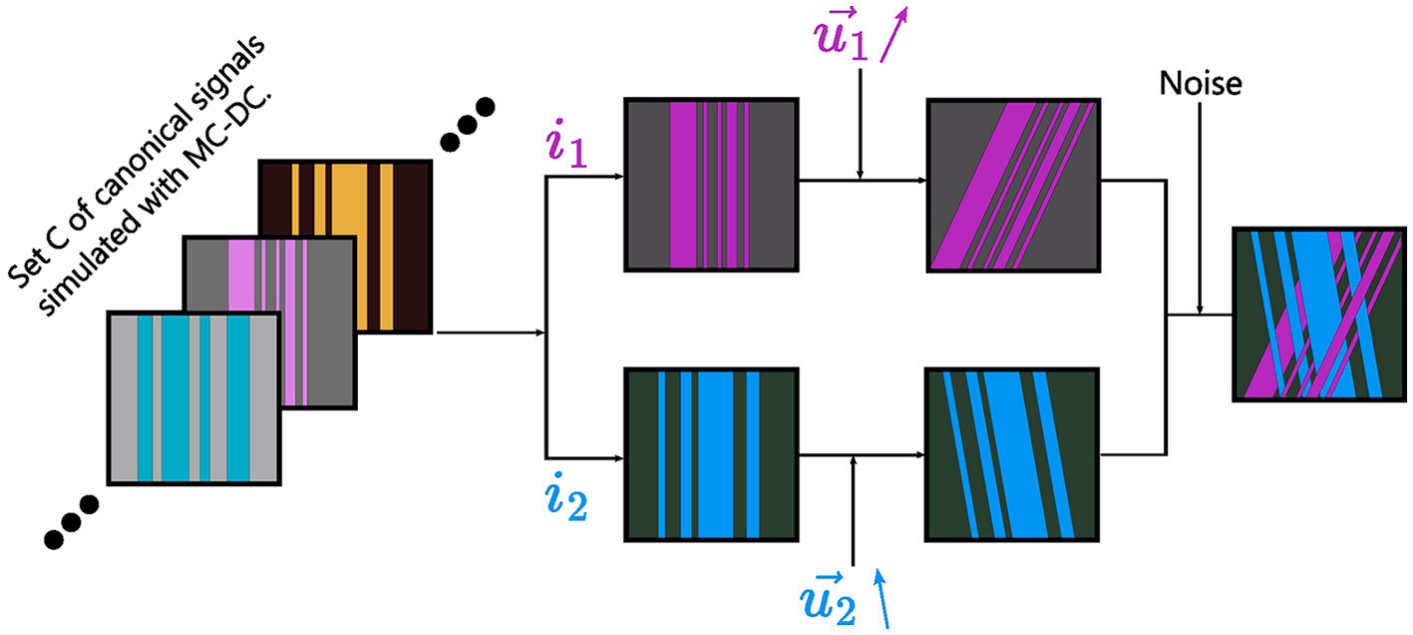
Graphical summary of the method used to generate synthetic data from Monte Carlo simulations computed with the open source MC-DC simulator. First, two atoms *i*_1_ and *i*_2_ are randomly selected among 50 canonical signals simulated with MC-DC. They are rotated along two random directions $\vec{{\text{u}}_{1}}$ and $\vec{{\text{u}}_{2}}$ with a minimal separation angle of 25°. Finally, the synthetic sample is obtained by linearly mixing the two rotated atoms and adding noise.

Then, for each Signal to Noise Ratio (SNR) ∈{100, 50, 40, 30, 20, 10}, 20 000 samples were generated using the following procedure, which is also summarized in Figure 2:

1. Two canonical signals *c*_*i*_1__ and *c*_*i*_2__ are chosen in *C*.
2. Two directions $\vec{u_{1}}$ and $\vec{u_{2}}$ are uniformly chosen, with a minimal angle of 25° between them, and *c*_*i*_1__ (resp. *c*_*i*_2__) is rotated along $\vec{u_{1}}$ (resp. $\vec{u_{2}}$ ).
3. The synthetic signal is computed: *y* = ν_1_*c*_*i*_1__+(1−ν_1_)*c*_*i*_2__, where ν_1_ is randomly selected in [0.5, 0.85] with uniform probability.
4. *y* is contaminated by noise following a Rice distribution (Rice, 1944; Gudbjartsson and Patz, 1995; Alexander, 2009) with the given SNR.

As commonly done in DW-MRI (Canales-Rodrguez et al., 2019), SNR was defined with respect to the signal value at *B* = 0s.mm^−2^, i.e. SNR = *s*(*B* = 0)/σ. However, this means that for higher b-values, for which the signal decreases, noise becomes stronger compared to the noiseless signal.

The CSD algorithm was applied to the synthetic samples and peaks were extracted from the reconstructed ODF. The response function used was an average of the 50 simulated signals. The estimations of the directions were also computed using SBSD for *n*_0_∈{4, 6, 8}. Moreover, estimation of the per-voxel per-direction impulse responses was performed using ground truth bundle orientations together with Equation 5, with λ = 10^−4^ for all settings explored. The results were then compared both to the ground truth as well as an estimation obtained via a SMT based algorithm. For the SMT algorithm, the per-voxel response function was reconstructed from the estimated λ_||_ and λ_⊥_ using $R\left(B,z\right)=\exp\left(-B\lambda_{||}z^{2}\right)\exp\left(-B\lambda_{⊥}\left(1-z^{2}\right)\right)$, which is the analytical model hypothesized by SMT (Kaden et al., 2016b). Both parameters (λ_||_ and λ_⊥_) were estimated by using all shells investigated in this work, i.e. *B*∈{2000, 3000, 4000, 5000, 10000}s.mm^−2^. For fair comparison, the latter estimation (one per voxel) was weighted with the ground truth mix coefficient ν_*k*_ for each axon bundle to obtain per-direction impulse responses.

### 3.2 Experience B: application on Human Connectome Project data

The Human Connectome Project (HCP) (Setsompop et al., 2013; Van Essen et al., 2013) provides data with the aim of improving the understanding of the human brain structure, function and connectivity. In order to assess the behavior of SBSD on real *in-vivo* data, the algorithm was run on 5 participants of the HCP Young Adult diffusion preprocessed dataset (Feinberg et al., 2010; Moeller et al., 2010; Setsompop et al., 2012; Xu et al., 2012; Milchenko and Marcus, 2013; Sotiropoulos et al., 2013). In order to ease the reproducibility of the results presented in this paper, no additional preprocessing was performed compared to the general (Jenkinson et al., 2002; Glasser et al., 2013) as well as diffusion specific (Andersson et al., 2003; Fischl, 2012; Jenkinson et al., 2012; Andersson and Sotiropoulos, 2015, 2016) preprocessings proposed by the HCP. Results for the orientation retrieval tasks were compared to results from the CSD and MSMT-CSD algorithms. Moreover, estimation of the per-voxel per-directions impulse responses were made using the MSMT-CSD directions together with Equation 5 for the b-value *B* = 3000s.mm^−2^ with 64 directions on the sphere. The MSMT-CSD directions were thresholded with a relative peak amplitude of 0.1. Once the per-directions impulses are estimated, any method can be used in order to estimate per-voxel per-directions descriptors. In order to present an example of derivation of such properties from the reconstructed impulses, a descriptor of the shape of the reconstructed impulses was computed using the following procedure: a diffusion tensor was fitted to the per-direction impulse responses estimated using Equation 5 with λ = 10^−4^. Then, similarly to the usual FA, an Anisotropy Index (AI) is computed as


$$AI_{k}=\sqrt{\frac{3}{2}\frac{{\left(\lambda_{k,1}-{\hat{\lambda }}_{k}\right)}^{2}+{\left(\lambda_{k,2}-{\hat{\lambda }}_{k}\right)}^{2}+{\left(\lambda_{k,3}-{\hat{\lambda }}_{k}\right)}^{2}}{\lambda_{k,1}^{2}+\lambda_{k,2}^{2}+\lambda_{k,3}^{2}}}$$

for each direction *k* at each voxel, where λ_*k*, 1_, λ_*k*, 2_andλ_*k*, 3_ are the eigenvalues of the diffusion tensor fitted to the estimated per-direction impulse for direction *k* and ${\hat{\lambda }}_{k}$ their mean. The resulting maps were compared to maps of standard FA. Along-tract AI was computed for the corpus callosum and the frontal aslant tract using the per-direction AI maps. This was then compared to along-tract FA obtained from the conventional FA map. The along-tract metrics were computed using the publicly available UNRAVEL Python package (Delinte, 2023), which assigns microstructural properties along a tract by examining the nearest angular peaks (Delinte et al., 2023), similarly to earlier works such as Chandio et al. (2020).

### 3.3 SH expansion computation

The SHs expansion of the signals were obtained via LSI with the regularization proposed in Descoteaux et al. (2007) for all experiences. The LSI was performed with a truncation including SHs up to order 8 for b-values *B* ≤ 5 000s.mm^−2^, and up to order 10 for *B* = 10 000s.mm^−2^ for SBSD and CSD with Dipy. For the MSMT-CSD and CSD run with MRtrix3 at a b-value *B* = 5 000s.mm^−2^ on *in-vivo* data, default parameters were used.

### 3.4 Algorithms implementations

The SBSD algorithm was implemented using Python 3.11 and makes ample use of the *Quaternionic* (Boyle, 2023a) and *Spherical* (Boyle, 2023b) packages. The SMT algorithm was performed with the implementation publicly available at https://github.com/ekaden/smt.

For applications on *in-vivo* data, the Python code was parallelized using the *dask* package (Rocklin, 2015). The source code of the implementation is available at https://github.com/cfuchs2023/SBSD_public (https://doi.org/10.5281/zenodo.10866169) together with the scripts used to generate the results presented in this work.

For Experience A (on synthetic data), CSD and ODF peaks extraction were realized with the Dipy package (Garyfallidis et al., 2014), in order to compare SBSD to an implementation of CSD in the same language. For Experience B (on *in-vivo* data), CSD, MSMT-CSD and ODF peaks extraction were performed with the C++ implementation available in MRtrix3 (Tournier et al., 2019), and the CSD response function was obtained using the “Tournier” algorithm (Tournier et al., 2013) while the MSMT-CSD response functions were computed using the “dHollander” algorithm (Dhollander et al., 2016, 2019).

## 4 Results

As the algorithm comprises two stages (see Figure 1), the performance of each stage is evaluated individually.

### 4.1 Experience A: validation on Monte Carlo simulations

#### 4.1.1 Fascicle orientation estimation

**Figure 3:**
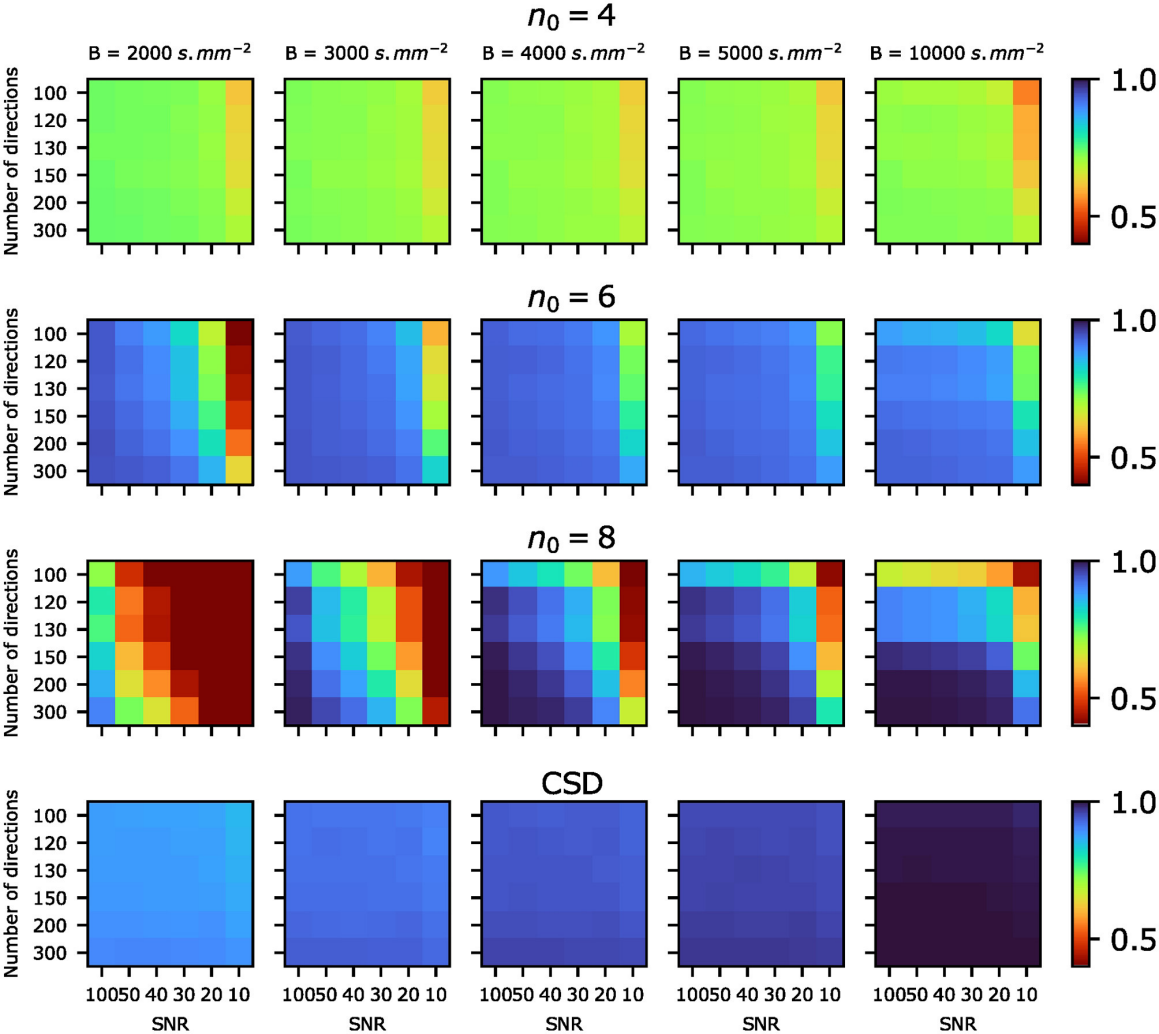
Proportion of fascicles for which the angular error was less than 10°, referred to as *accuracy*. The algorithm was run on the same synthetic data with different choices for *n*_0_, i.e., the degree of the coefficients used for direction estimation. For each of this choice, the accuracy is plotted for different values of SNR and number of directions of the acquisition schemes.

Figure 3 shows the proportion of fascicles for which an error of less than 10° was achieved, which will be referred to as the accuracy of the algorithm. Performing the orientation estimation with *n*_0_ = 4 fails to achieve accuracy higher than 0.8 even for b-value *B* = 10 000s.mm^−2^, SNR = 100 and 300 directions. However, it is also the choice most robust to noise, consistently achieving accuracy greater than 0.7 for SNR ≥20. The highest degree (*n*_0_ = 8) is the choice that attains the highest accuracy of 1, for settings of high SNR = 20 together with 250 directions at b-value *B* = 10 000s.mm^−2^. Although such acquisition schemes are not usual in clinical settings, it is notable that the acquisition scheme used by the Human Connectome Project (HCP) in the MGH HCP Adult Diffusion dataset has a shell of 256 directions at b-value *B* = 10 000s.mm^−2^. However, the accuracy at *n*_0_ = 8 quickly diminishes at SNR 10, and reliably outperforms *n*_0_ = 6 only for b-values *B*≥4 000s.mm^−2^. Degree 6 outperforms degree 8 at SNR 10 for all number of directions, and for some directions at SNR 20 and 30. It also significantly outperforms degree 8 at clinically usual b-value *B* = 3 000s.mm^−2^, achieving accuracy between 0.8 at SNR 20 and 0.95 at SNR 30.

**Figure 4:**
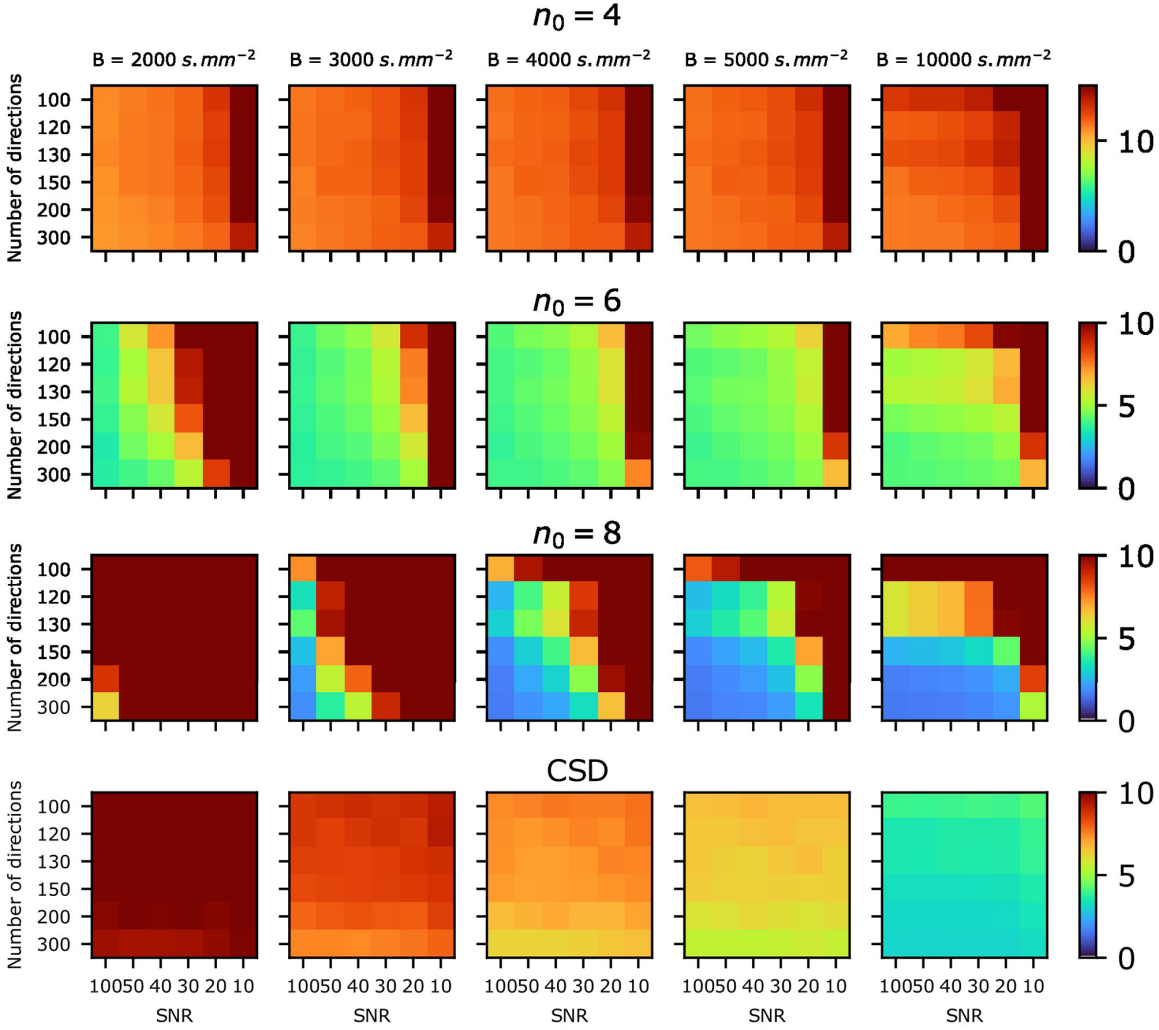
Average angular error (in degrees) over all fascicles plotted for various choices of *n*_0_ for the SBSD algorithm as well as CSD, for various values of SNR and number of directions on the sphere.

Figure 4 shows the average angular error over all fascicles for various choices of *n*_0_ as well as values of SNR and number of directions on the sphere. Less than 10° average error is consistently achieved for *n*_0_ = 6, while the lowest average error is achieved for *n*_0_ = 8 for combinations of sufficiently high SNR, b-value and number of directions. Notably, *n*_0_ = 8 achieves significantly lower average angular error than CSD in favorable settings, while *n*_0_ = 6 achieves a lower improvement over CSD but on a larger set of SNR, b-values and number of directions. *n*_0_ = 4 achieves lower or similar average angular error compared to CSD at b-values *B* = 3 000 and 2 000s.mm^−2^.

In *in-vivo* data a deviation is expected from the formulation given in Equation 4. Specifically, it is expected that the performance will drop more significantly the higher the degree, since they are more sensitive to noise and errors in the SH expansion computation.

#### 4.1.2 Reconstruction of per-voxel per-direction impulse responses

**Figure 5:**
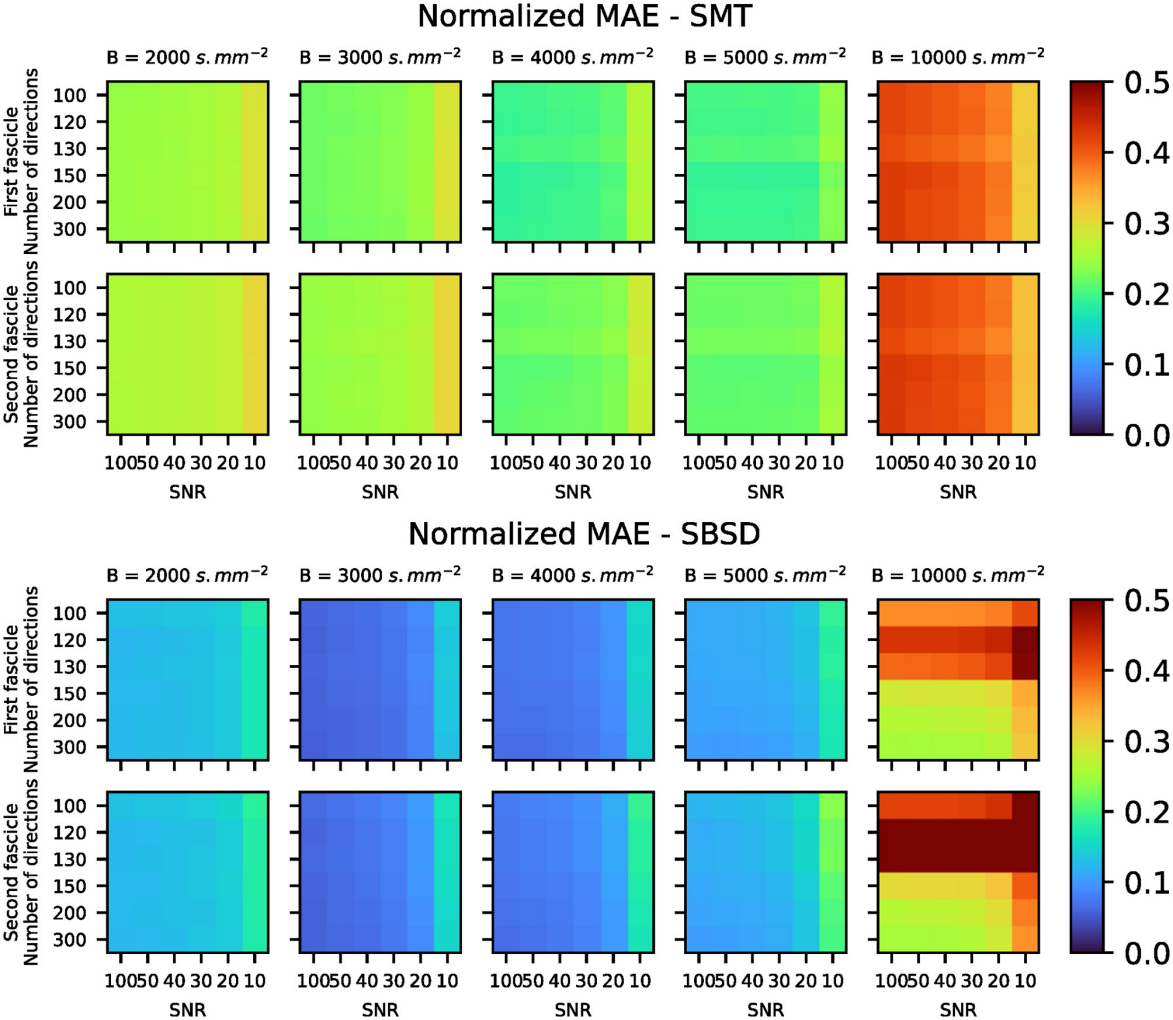
Mean Absolute Error (MAE) of the estimated impulse responses compared to the ground truth. For each synthetic voxel and each fascicle, the error vivo normalized by the mean of the ground truth impulse response in order to compare results over a wide range of b-values, for which the signal changes scale. As SMT only estimates one impulse response per voxel, it was weighted with the ground truth mix coefficient associated with each fascicle for a fair comparison with the ground truth impulse responses.

**Figure 6:**
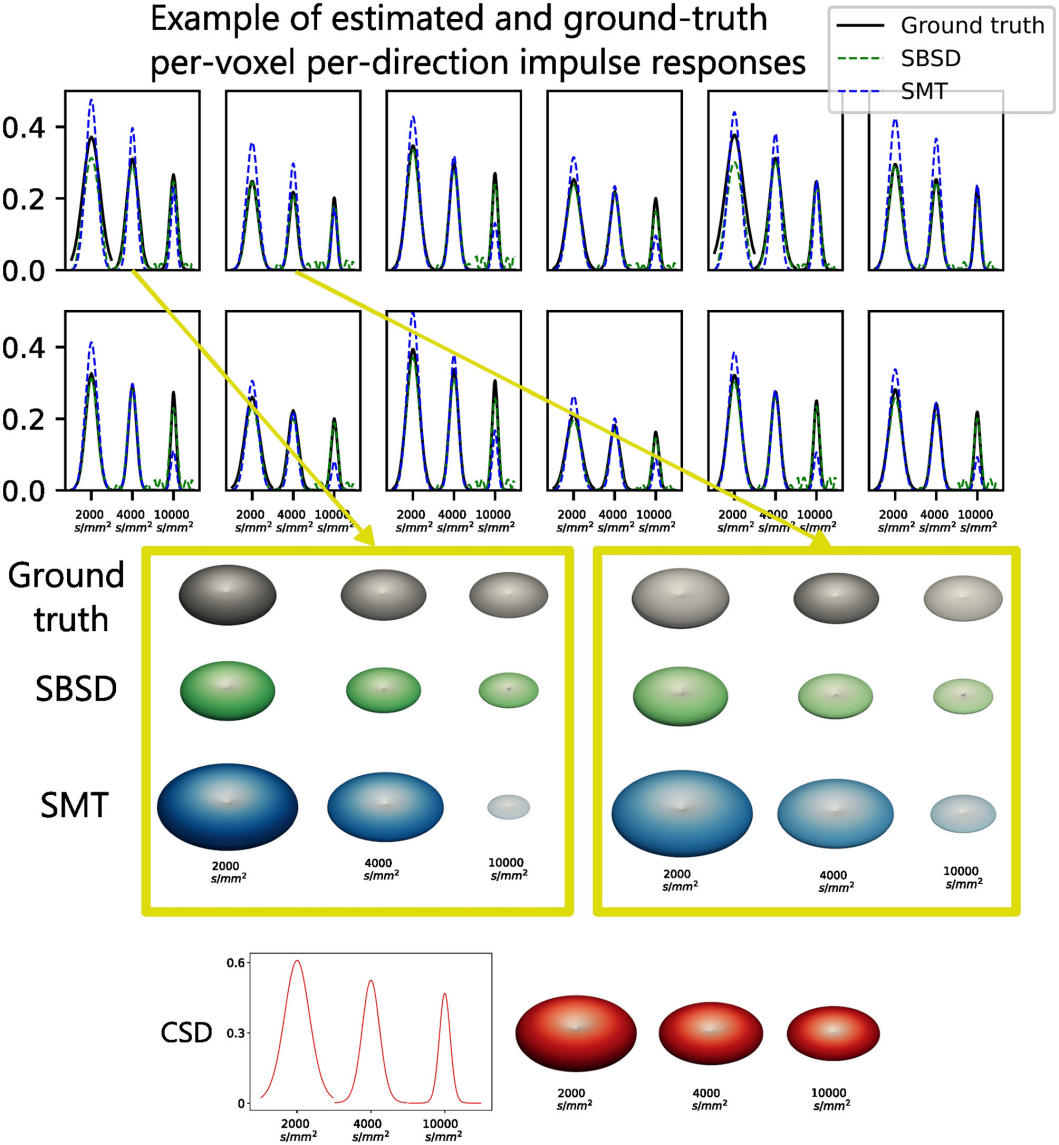
Examples of impulses estimated by using either Equation 5 (in green) or SMT (in blue) on synthetic data generated following the procedure described on Figure 2 and in Section 3.1. The SMT response was weighted by the ground truth ν_*k*_ coefficients to obtain per-direction impulse responses. The Monte-Carlo simulated ground truth is shown in black. Below, the impulse response used by CSD, which is an average of all 50 canonical signals used to generate the synthetic data, is shown in red.

Figure 5 show the Mean Absolute Error (MAE) of the estimated impulses using either Equation 5 or SMT compared to the ground truth, while Figure 6 shows examples of estimated impulse responses. It is notable that the proposed method outperforms SMT on all settings of SNR and number of directions for b-values *B*∈{2 000, 3 000, 4 000, 5 000}s.mm^−2^ in terms of MAE. Both methods exhibit little sensitivity to the number of sampling points of the signal in the range studied (from 100 to 300 sampling points, i.e., from 50 to 150 acquisition gradients) for b-values *B* ≤ 5 000s.mm^−2^. as well as a breakdown of performance at *B* = 10 000s.mm^−2^. This might seem counter-intuitive and is investigated thoroughly in the Discussion section. Figure 6 shows that estimating the impulses using Equation 5 leads to ringing at both ends of the estimation (i.e., close to the poles of the 2-sphere), while SMT does not provide accurate estimation over the wide range of b-values used.

### 4.2 Experience B: application to HCP data

#### 4.2.1 Fascicle orientation estimation

**Figure 7:**
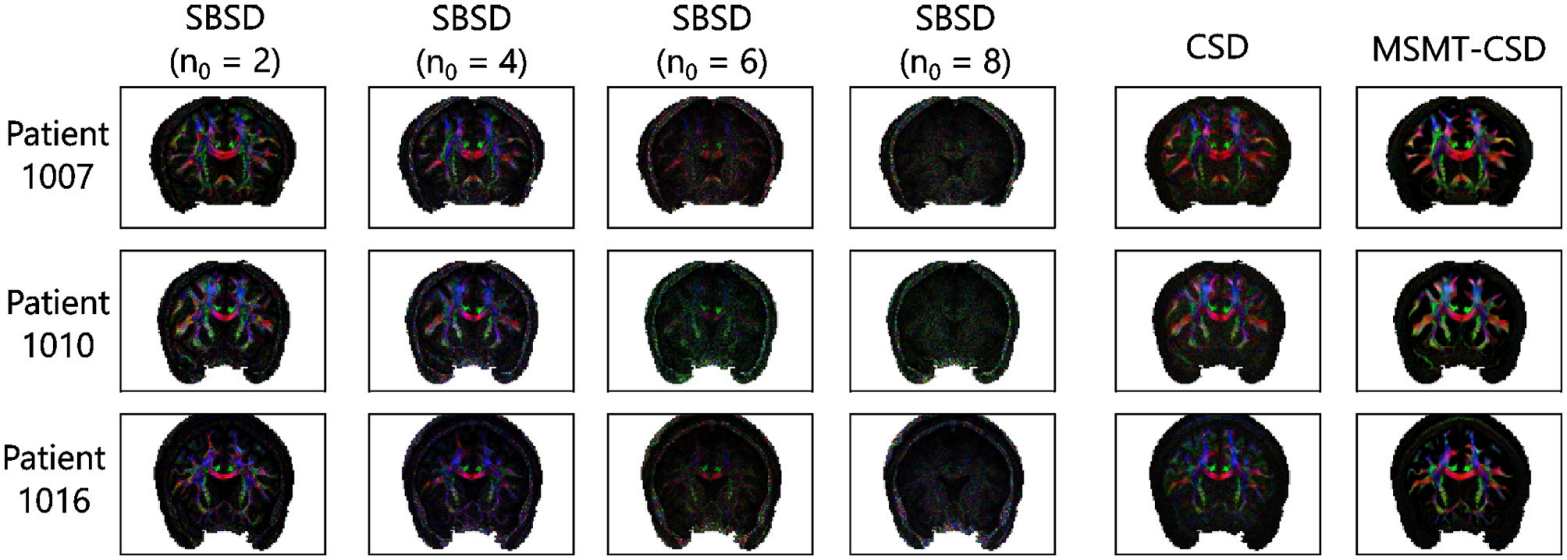
Results of the estimation of fibers orientation performed on three patients of the HCP at b-value *B* = 5, 000s.mm^−2^ with 128 directions on the sphere. The estimated directions are weighted by the modulus of the associated $\nu_{k}C_{k,0}^{n_{0}}$ (green highlight in Figure 1) estimated by SBSD. The results are visualized with the usual RGB convention where red corresponds to the left-right axis, green to the anterior-posterior axis and blue to the inferior-superior axis.

**Figure 8:**
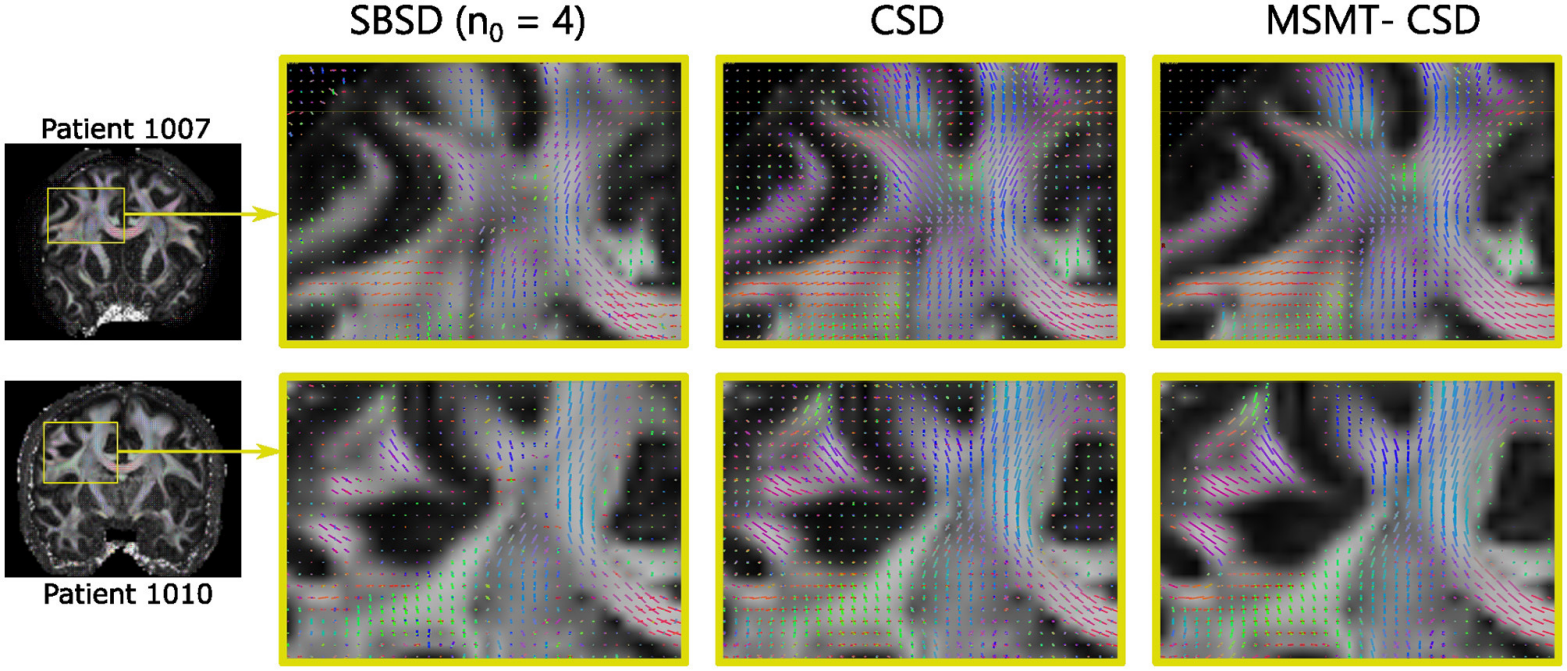
Zoom on an arbitrary brain region for the two first HCP patients shown on Figure 7, showing estimated fascicles orientations in an arbitrary region including parts of the cingulum, corpus callosum, corticospinal tract and arcuate fasciculus. The estimated orientations are peaks of the ODF for CSD and MSMT-CSD. The background is a fractional anisotropy map obtained with the DTI implementation available in Mrtrix3.

The SBSD algorithm was run on five patients (1007, 1010 and 1016, 1019 and 1031) of the MGH HCP Adult Diffusion dataset and results for the first three are presented on Figure 7 while Figure 8 shows additional details for the first two patients.

Figure 7 shows that on *in-vivo* data, SBSD is likely able to estimate the orientation of single bundles for *n*_0_∈{2, 4} : the corpus callosum is red-colored and the cingulum green-colored. Although the corpus callosum and corticospinal tract can still be perceived for *n*_0_ = 6, the results are largely contaminated by noise. For *n*_0_ = 8, the ventricles are still visible but even large structures like the cingulum and corpus callosum are indistinguishable from the gray matter. Figure 8 shows a zoom for the two first patients (1007 and 1010) in the coronal view on a region containing parts of the corpus callosum, cingulum, cerebro spinal tracts and arcuate fasciculus. The results from the proposed SBSD algorithm are compared to those obtained with CSD and MSMT-CSD. In regions occupied by a single tract, such as in the corpus callosum or cingulum, results of SBSD are broadly comparable to CSD and in agreement with MSMT-CSD. However, SBSD often only estimates one of the orientation correctly and fails to retrieve the other, such as in the area where the arcuate fasciculus crosses projections from the corpus callosum.

#### 4.2.2 Reconstruction of per-voxel per-direction impulse responses

**Figure 9:**
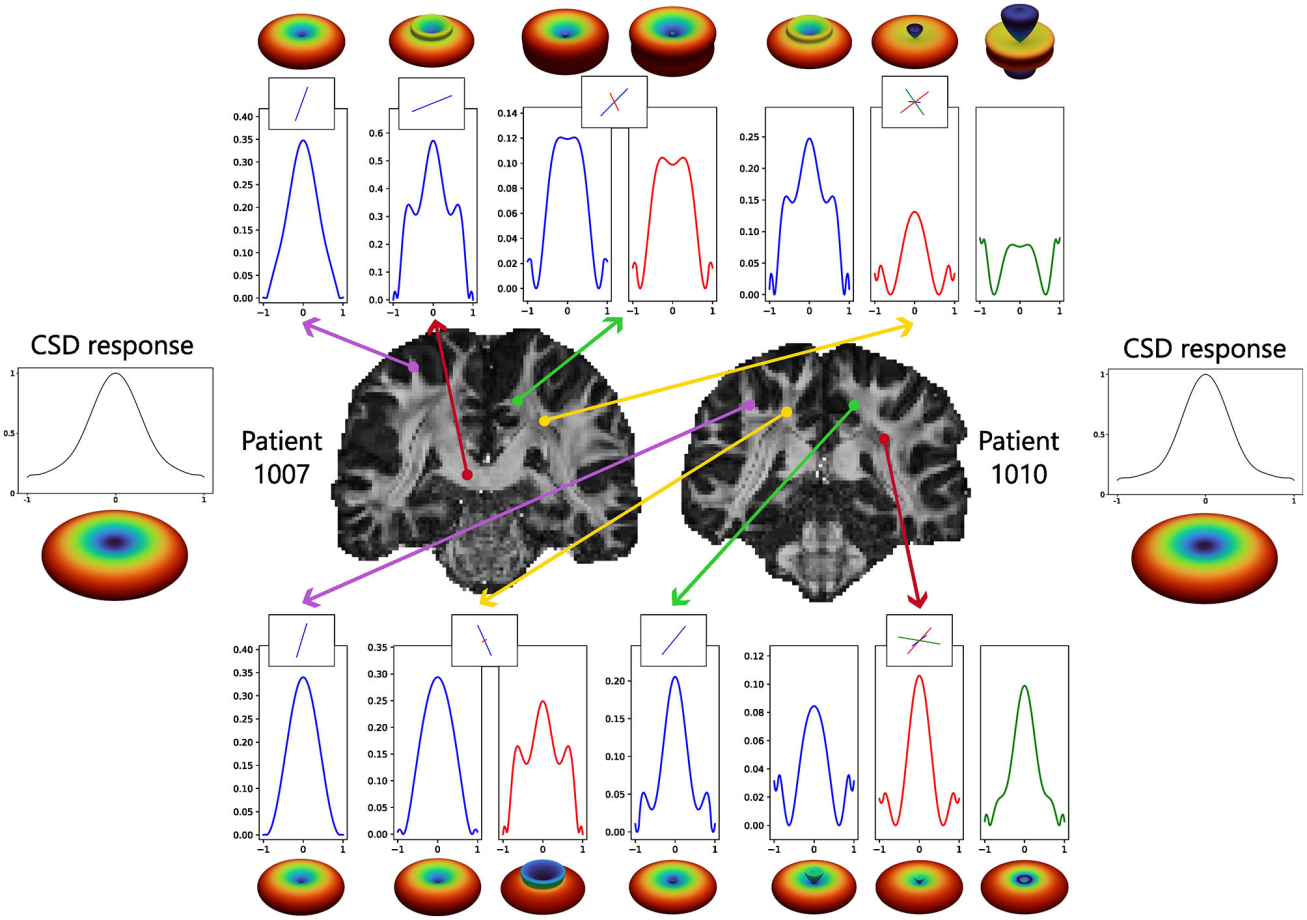
Examples of per-voxel per-directions estimated impulse responses using MSMT-CSD ODF peaks at a b-value *B* = 3, 000s.mm^−2^ with 64 directions on the sphere following Equation 5. Below or above each 1D plot, a 3D visualization of the estimated impulse response is shown. Together with the impulses, the directions of the MSMT-CSD peaks projected in the plane of the slices are shown as crossing sticks. As the peaks were thresholded according to their amplitudes, there can be one, two or three directions per voxel. These ℓ_2_ normalized directions were used to estimate the impulse responses.

**Figure 10:**
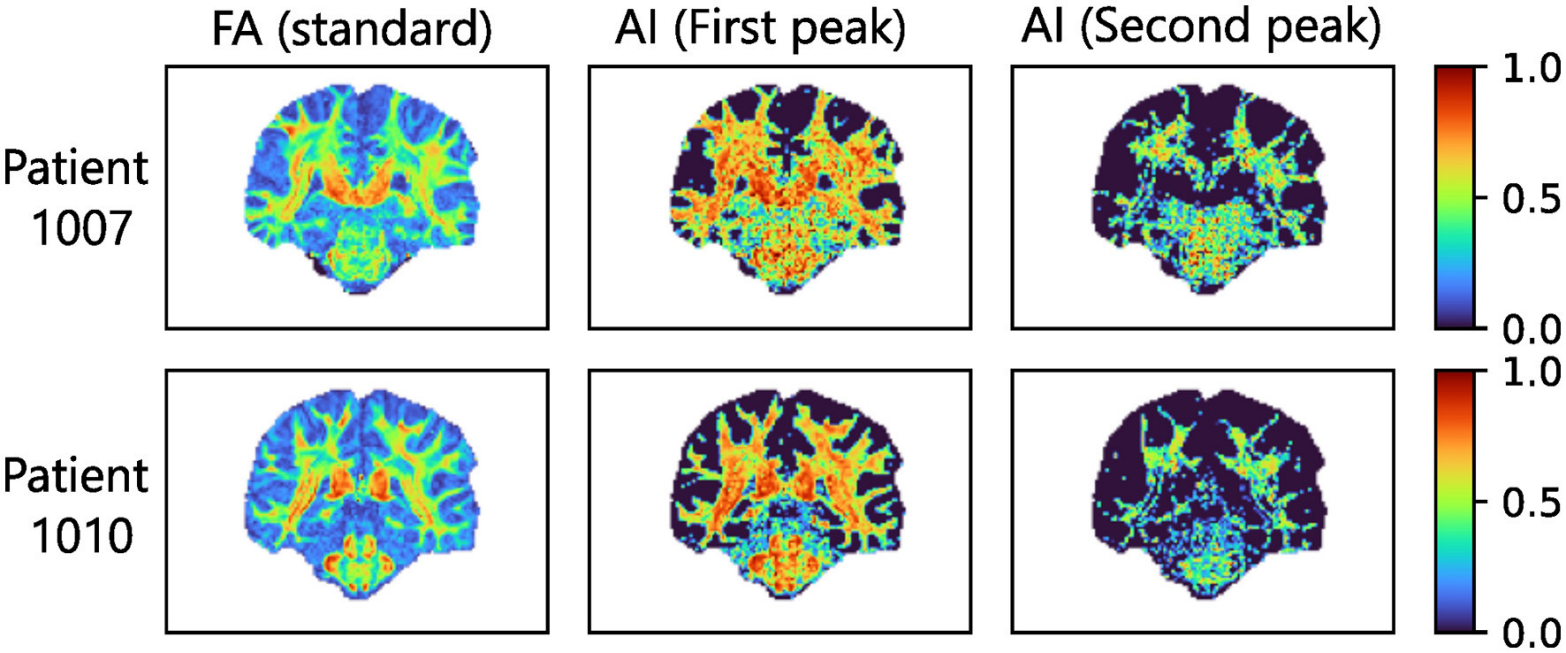
FA map obtained by running DTI with MRtrix on the diffusion signal (left column) as well as AI maps derived from impulse responses estimated using Equation 5 together with the first (resp. second) peaks of the MSMT-CSD ODF (middle (resp. right) columns) at a b-value *B* = 3, 000s.mm^−2^ with 64 directions on the sphere. For the latter two, no responses were estimated in voxels where no MSMT-CSD peaks exceeded the threshold, resulting in an AI of 0 in those voxels.

**Figure 11:**
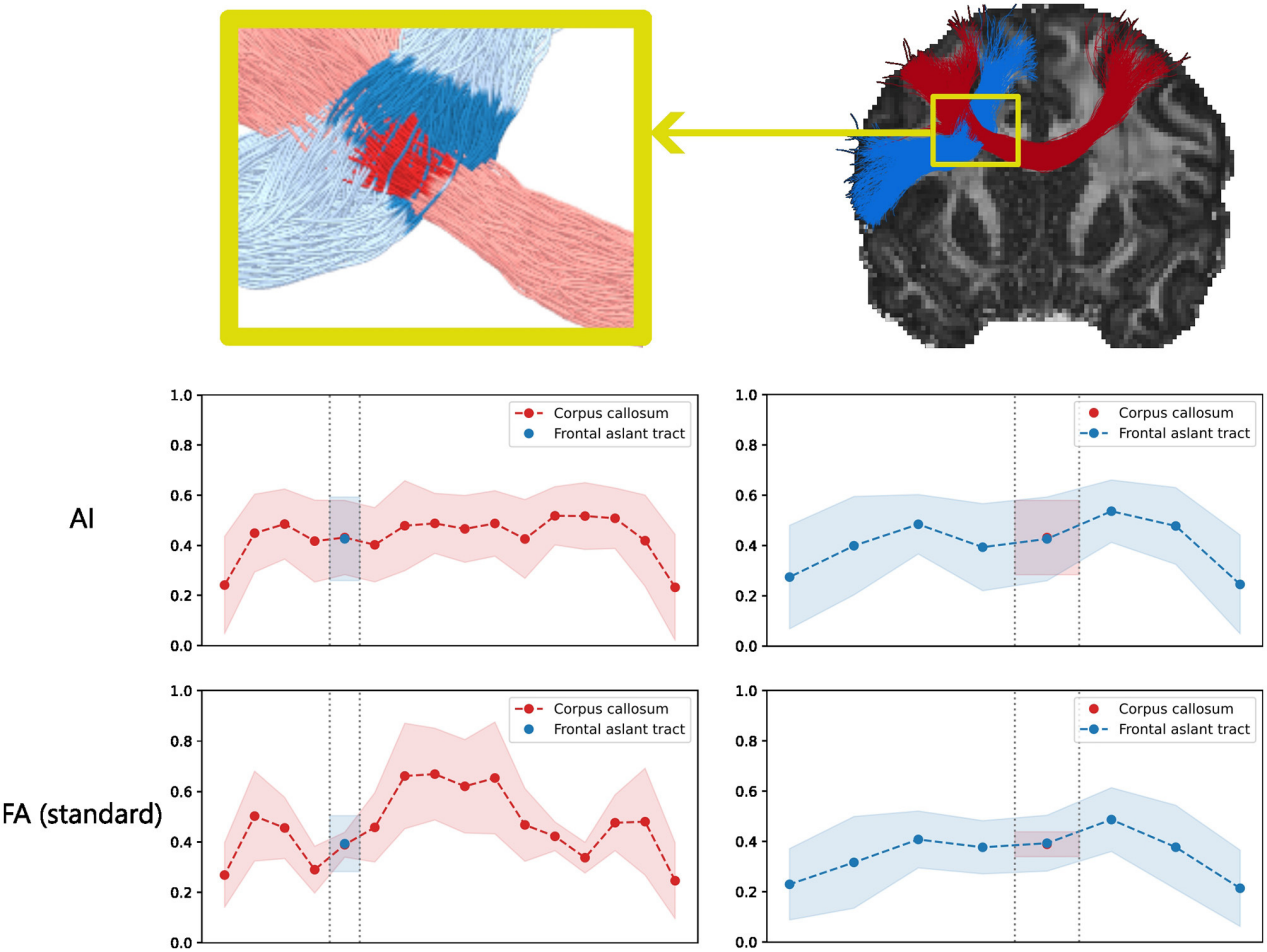
Along-tract AI (**first row** of graphs) for the corpus callosum and frontal aslant tract for patient 1007, derived from impulse responses estimated following Equation 5. The **second row** of graphs shows the results using the standard FA map.

Figure 9 shows examples of estimated impulse responses for two patients of the HCP at different locations in the brain. Similarly to the results on synthetic data, ringing is often observed at the ends of the reconstructed impulses. Figure 10 shows the standard FA computed from the diffusion signal, as well as per-directions AI maps computed from the estimated per-direction impulses. The per-directions AI maps exhibited at least one direction with high AI in the regions where the arcuate fasciculus and corticospinal tract cross projections from the corpus callosum, where the standard FA drops significantly. Figure 11 shows along-tract FA using maps of standard FA as well as along-tract AI using per-direction AI maps. Similarly to Figure 10, the per-directions AI are generally lower than the standard FA. Moreover, for the corpus callosum, the standard FA drops significantly near and within the crossing with the frontal aslant tracts, while the per-direction AI remains close to constant. Both metrics show a significant decrease when approaching the gray matter, i.e. at the ends of the tract.

## 5 Discussion

### 5.1 Orientation retrieval on Monte Carlo simulations derived data

The proposed algorithm was first tested on synthetic data obtained with Monte Carlo simulations in Experience A. SBSD achieved lower average angular errors for direction estimation in a simplified synthetic setting at high SNR (≥20) compared to CSD, as shown in Figures 3, 4. This was likely due to the use of a fine grained dictionary in the SBSD algorithm, i.e., using a high angular resolution sampling of the sphere, which was made possible thanks to the low computational complexity of the OMP. However, the relaxation of hypothesis about the response function also induced a greater sensitivity to errors in the computation of the SH expansion of the signal, mainly driven by noise and number of directions on the sphere (Figures 3, 4). Recently, efforts have been made to develop spherical Fourier transform algorithms dedicated to axially symmetric signals (Bates et al., 2016) or methods more resistant to noise for SH expansion computation (Tarar and Khalid, 2021), but no clear state-of-the-art method significantly improving over the least squares inversion proposed in Descoteaux et al. (2007) has emerged yet. Overall, this experience validated the ability of the SBSD algorithm to accurately estimate the directions of two crossing fascicles of parallel fibers, provided that both the number of directions on the shell and SNR are sufficiently high.

### 5.2 Impulse responses estimation on Monte Carlo simulations derived data

As SMT hypothesizes that all axon bundles have the same response regardless of their orientation, the statistical independency of the diffusion properties of the two crossing bundles in the synthetic data made it a challenging task for SMT. Moreover, SMT uses an analytical model for the response *R* of axon fascicles such that *ln*(*R*) is linearly dependent on *B*. This is not the case in *in-vivo* data (Jensen et al., 2005) nor in the Monte-Carlo simulated data used for Experience A, which explains why SMT cannot accurately estimate the impulse responses over a wide range of b-values, as well as its significant drop in performance for *B* = 10 000s.mm^−2^. Conversely, since the proposed method does not need to assume a generative model for the impulse response, it was able to recover per-direction impulse responses accurately for all b-values *B* ≤ 5 000s.mm. Ringing was often observed (see Figure 6) at both ends of the impulse responses estimated following Equation 5, which is linked to core properties of the SHs. Indeed, SHs of order 0 are proportional to Legendre polynomials. Therefore, the estimations are in fact polynomials of the same degree as the maximum degree used for SHs coefficients computation. However, the ground truth is exponential in nature and peaked around *z* = 0. Consequently, the truncature at degree 8 or 10 induced ringing in the reconstructed impulses. This is particularly challenging for settings of high b-values, because the relative increase in noise complicates the estimation of SHs expansion while the degree needed to accurately represents the signal increases. It explains the significant drop in performance observed for the b-value *B* = 10 000s.mm^−2^. Moreover, the estimations were found to systematically undershoot the peak at *z* = 0. Depending on the method used to link those impulses to diffusion properties, this could lead to systemic bias in the estimation of parameters such as longitudinal diffusivity. Overall, the proposed method for estimating per-voxel per-direction impulse responses was found to be robust and accurate on synthetic data generated following the procedure described on Figure 2 and in Section 3.1., significantly outperforming SMT in terms of MAE (Figure 5) for all settings of SNR and number of directions for a clinically realistic range of b-values *B*∈{2 000, 3 000, 4 000, 5 000}s.mm^−2^. However, one should note that the ODF in the synthetic data were indeed sum of Dirac impulses on the sphere, with one peak per direction.

### 5.3 Axon bundles orientation prediction on *in-vivo* data

SBSD was then run on the reduced sampling of the HCP Young Adult diffusion dataset comprising five patients (1007, 1010, 1016, 1019 and 1031) (Experience B) and results were shown for the first three patients with more details being provided for the first two. Visually, results of orientation estimation of SBSD were broadly similar to those of CSD in regions where a single axon bundle was present, with both methods agreeing with MSMT-CSD (Figures 7, 8). Although SBSD, compared to CSD, exhibited much smaller magnitude for peaks in regions where no fibers were expected such as the ventricles, SBSD performed noticeably worse in regions comprising multiple crossing fiber bundles, failing to retrieve the expected orientations in a significant number of such voxels. This was coherent with the findings of greater noise sensitivity highlighted during Experience A. Moreover, it is likely that deviations from the expected model summarized in Equation 4, such as tortuosity of axon bundles which invalidates the hypothesis of axial symmetry of the signal, also played a role, although CSD also hypothesizes the axial symmetry of its response function.

### 5.4 Per-direction impulse responses estimation

Since the estimation of the per-direction impulse responses corresponding to axon bundles can be performed with any orientations estimate (Equation 5), such an estimation was computed using the peaks of the MSMT-CSD ODF. Although *in-vivo* ODF are often assumed to be sparse and peaked (Canales-Rodrguez et al., 2019), they are not sum of Dirac impulses because the axons are not perfectly parallel and have tortuosity. Therefore, the estimated impulses are likely flatter than the ground truth because they need to account for the spread of the ODF around the Dirac peaks modeled by the $U_{{\text{v}}_{k}}^{n}$ in Equation 5. Both those factors mean that depending on the method used to compute descriptors from the impulse responses, bias could be introduced by the proposed method. In Figure 11, the standard FA decreases sharply in regions where multiple tracts crosses, which is a well-known property of the DTI model which measures the anisotropy of the diffusion signal on a voxel wise scale without considering crossings of axon bundles. Conversely, for the per-direction AI maps, at least one peak was associated with a high AI value in those regions and the along-tract AI in Figure 11 was close to constant for the corpus callosum, except at the ends of the tracts where the white matter reaches the gray matter. This is an argument showing that the proposed method at least partially disentangles diffusion properties of crossing axon bundles, although the estimated impulse responses include information about the ODF. This also partially explains the decreases of the per-direction AI close to the gray matter, because the ODF becomes more spread out which flattens the estimated impulse responses. This also means that linking the estimated impulse responses to properties such as axonal density is not straightforward.

### 5.5 Summary of the contribution

Given the results obtained on *in-vivo* data, the use of SBSD for orientation estimation in clinical settings is not recommended without further improvements. However, the work presented in this paper could open the door to a new family of spherical deconvolution algorithms, allowing for more detailed characterization of axon bundles, and is of interest for people developing methods for this particular problem. Indeed, although blind or ℓ_0_ penalized methods for solving spherical deconvolution problems have been proposed (Canales-Rodrguez et al., 2019), our contribution is twofold. First, the blind framework proposed does not need prior information about a generative model of the white matter response, but only an assumption about its axial symmetry. Second, our algorithm effectively leverages ℓ_0_ penalization in a fast and efficient way. Both of these contributions arise from the theoretical formulation of Equation 4, which gives a sparse representation of the SH expansion of the DW-MRI signals using a dictionary of highly uncorrelated atoms which are not dependent on the observations, but only on mathematical properties of the SHs and D-Wigner functions. This equation was derived under the explicit assumption that the observed DW-MRI signal is a sparse sum of axially symmetric signals, as is hypothesized in Daducci et al. (2014) or the NNLS-BSS-EBIC method presented in Canales-Rodrguez et al. (2019). Additionally, using the peaks of MSMT-CSD, maps of per-direction AI were derived from the per-direction impulse responses estimated using Equation 5, which showed resemblance to the brain anatomy (Figures 10, 11). Therefore, the second stage of the proposed algorithm (Figure 1) in conjunction with MSMT-CSD or another robust direction estimation method could be of interest to clinicians in order to study along-tract variation of metrics between populations.

### 5.6 Future works

We believe SBSD to be the most straightforward approach to exploit the core ideas exposed in the Theory section. This means there is likely room for building upon the algorithm exposed in this work; future works could include leveraging information from multiple shells instead of one, and using SBSD in a multi tissues framework. Moreover, as OMP uses a least squares problem during its greedy reconstruction of the sparse representation, it was natural to also solve a least squares problem for SH expansion estimation. However, taking into account the non-Gaussian nature of noise in magnitude reconstructed DW-MRI images would lead to other formulations of the optimization problem used to estimate the SH expansion associated with each bundle (Alexander, 2009).

## 6 Conclusion

This work proposed an algorithm of blind spherical deconvolution called Sparse Blind Spherical Deconvolution (**SBSD**), which, instead of necessitating exact knowledge of a response function, only assumes its axial symmetry. This relaxation allows for the retrieval of the individual signals arising from each bundle in addition to the orientations of the bundles. On the orientation retrieval task, SBSD outperformed the CSD algorithm in some settings of high SNR (≥20) in a synthetic and simplified test case, but was more sensitive to noise and errors in the computation of the SH expansion of the signal. On real *in-vivo* data, SBSD performed similarly to CSD, in voxels where one fiber population associated with a given orientation was dominant. However, in voxels where roughly similar fiber populations crossed, SBSD was found to be more prone to errors or straight failures than CSD, which was likely caused by SBSD's greater sensitivity to noise and SH expansion computation errors as well as deviation from the expected axial symmetry of impulse responses due to axonal tortuosity and fiber fanning. Both methods were outperformed by MSMT-CSD, which necessitates multi-shell data. Since the retrieval of signals arising from each bundle is agnostic to the method used to estimate the relevant orientations, per-direction impulse responses were estimated using peaks from the MSMT-CSD ODF. Maps of per-direction metrics computed using an approach similar to the standard fractional anisotropy derived from the estimated per-direction impulse responses showed resemblance to brain anatomy and remained close to constant in the studied regions of crossing fascicles along the studied tracts. Therefore, this method proved its potential for the derivation of per-voxel per-direction metrics, and potential improvements to spherical deconvolution algorithms by removing the need for an explicit response function for the white matter, thus enabling the retrieval of additional information about axon bundles.

## Data availability statement

Publicly available datasets were analyzed in this study. This data can be found here: https://db.humanconnectome.org/.

## Author contributions

CF: Conceptualization, Methodology, Writing – original draft, Writing – review & editing, Data curation, Formal analysis, Software. QD: Conceptualization, Data curation, Investigation, Writing – review & editing. ND: Conceptualization, Writing – review & editing. MD: Investigation, Writing – review & editing. BM: Funding acquisition, Project administration, Supervision, Writing – review & editing.

## Appendix A

### Angles conventions

Spherical coordinates are defined according to the common mathematical convention where θ is the colatitude [i.e. the oriented angle $\left(\vec{u_{Z}},\vec{OM}\right)$] and Φ the longitude [i.e., the oriented angle $\left(\vec{u_{x}},{\vec{OM}}_{z}\right)$ where ${\vec{OM}}_{z}$ is the projection of $\vec{OM}$ in the plane *z* = 0]. For Euler angles, the common *zyz* convention is used together with the notations α, β, γ : γ is the first rotation, around $\vec{u_{z}}$, then β around $\vec{u_{y}}$ and finally α around $\vec{u_{z}}$. Consequently, the rotation of a signal axysymmetric around $\vec{u_{z}}$ is only dependent on α and β.

### Spherical harmonics and D-Wigner functions

Multiple conventions can be found in the literature for the definition of spherical harmonics (SHs) or D-Wigner functions. In this work, the following definition is used for spherical harmonics:

$$\begin{array}{l} \;\forall n\in \mathbb{N}\text{,}\forall m\in \left[\left|-n,n\right|\right],\\ Y_{n}^{m}\left(\theta ,\Phi \right)=\sqrt{\frac{\left(2n+1\right)\left(n-|m|\right)!}{4\pi \left(n+|m|\right)}}P_{n}^{\left|m\right|}\left(cos\left(\theta \right)\right)e^{im\Phi }, \end{array}$$

where

$$P_{n}^{m}\left(x\right)={\left(-1\right)}^{m}{\left(1-x^{2}\right)}^{\frac{m}{2}}\frac{{\text{d}}^{m}P_{n}\left(x\right)}{\text{d}x^{m}}$$

are the associated Legendre polynomials and

$$P_{n}\left(x\right)=\frac{1}{2^{n}n!}\frac{{\text{d}}^{n}}{\text{d}x^{n}}{\left(x^{2}-1\right)}^{n}$$

the Legendre polynomials. SHs form an orthonormal basis of the Hilbert space *L*^2^(*S*^2^) and therefore, any *f* ∈ *L*^2^(*S*^2^) can be defined with its expansion over the SHs $\forall \omega \in S^{2},f\left(\omega \right)=\overset{\infty }{\underset{n=0}{\sum }}\overset{n}{\underset{m=-n}{\sum }}f_{n}^{m}Y_{n}^{m}\left(\omega \right)$. The coefficients $f_{n}^{m}$ are defined by $f_{n}^{m}=\int_{\omega \in S^{2}}f\left(\omega \right)\bar{Y_{n}^{m}\left(\omega \right)}d\omega$. Note that the antipodal symmetry of diffusion signals implies that all $f_{n}^{m}=0$ for *n* odd.

In this work, the following definition is used for D-Wigner functions

$$\begin{array}{l} \begin{array}{c} \forall n\in \mathbb{N}\text{,}\forall m\in \left[\left|-n,n\right|\right]\text{,}\forall q\in \left[\left|-n,n\right|\right]\text{,}\\ D_{n}^{m,q}\left(\alpha ,\beta ,\gamma \right)=e^{-jm\alpha }d_{n}^{m,q}\left(\beta \right)e^{-jq\gamma }, \end{array} \end{array}$$

with the d-Wigner functions $d_{n}^{m,q}$

$$\begin{array}{l} \begin{array}{c} d_{n}^{m,q}\left(x\right)=j^{m-q}\frac{sin{\left(x\right)}^{q-m}{\left(1+cos\left(x\right)\right)}^{m}}{2^{n}{\left(\left(n+m\right)!\left(n-q\right)!\right)}^{1/2}}{\left[\frac{\left(n-q\right)!}{\left(n+q\right)!}\right]}^{1/2}\\ \frac{{\text{d}}^{n+q}}{\text{d}cos{\left(x\right)}^{n+q}}\left[{\left(cos\left(x\right)-1\right)}^{n+m}{\left(cos\left(x\right)+1\right)}^{n-m}\right]. \end{array} \end{array}$$
